# Management of Laterally Luxated Primary Teeth: A Case Report

**DOI:** 10.7759/cureus.35218

**Published:** 2023-02-20

**Authors:** Simran Das, Nilima R Thosar, Monika Khubchandani, Nishi S Malviya, Rutuja Ragit

**Affiliations:** 1 Pediatric and Preventive Dentistry, Sharad Pawar Dental College and hospital, Datta Meghe Institute of Higher Education and Research, Wardha, IND; 2 Pediatric and Preventive Dentistry, Sharad Pawar Dental College and Hospital, Datta Meghe Institute of Medical Sciences (Deemed to be University), Wardha, IND

**Keywords:** traumatic dental injuries (tdis), flexible splinting, lateral luxation, deciduous teeth, pediatric dental trauma

## Abstract

Lateral luxation can be defined as the traumatic displacement of a tooth in a direction other than axial. The current case report describes the use of composite resin splinting to treat laterally luxated primary maxillary central incisors which resulted in an anterior cross bit. A 5-year-old boy reported to the clinic complaining of pain and mobile front teeth that is his primary right and left central incisors were laterally luxated. After a complete clinical examination and radiographic evaluation, the teeth were repositioned and stabilized for 4 weeks by splinting the laterally luxated tooth to the adjacent teeth. Follow-up examinations revealed that the tissues had healed well and that the corresponding central permanent incisor was healthy and unaffected. This case study highlights the significance of prompt diagnosis, effective treatment, and routine follow-up of traumatized teeth because they may have an adverse effect on both dentitions and “Oral Health-Related Quality of Life”. When feasible, conservative treatment should be considered because it may be more suitable in some circumstances.

## Introduction

Traumatic dental injury (TDI) is a condition that is highly prevalent and seen worldwide, according to epidemiological studies [1]. Data show that one-third of adults and one-fourth of school-aged children have had dental trauma [1]. Due to their active participation in sports and games, males often report more severe tooth injuries than females [2]. The most affected are maxillary central incisors, which account for 37% of cases, and mandibular central incisors teeth 18%, mandibular teeth 12%, and followed by mandibular teeth 4% [1-2]. The greatest incidence of trauma to the primary teeth occurs at 2 to 3 years of age when motor coordination is developing primary teeth are particularly susceptible to luxation (displacement) injuries, which account for 21%-81% of all TDI [3]. The most serious TDI, intrusion, affects 0.5% to 1.9% of all patients [3]. Due to severe damage to the periodontal ligament and pulp fibers, it has the worst prognosis [3].

Lateral luxation is another form of TDI that has damage to one of the root surfaces and has comparable effects to incursion [4]. To lessen and prevent future injuries-related consequences after TDI, therapy must be administered as quickly as possible, ideally within the first hour [5]. Repositioning and splinting are urgent treatments for teeth with incursion and/or lateral luxation damage. After 2 weeks, the endodontic treatment is performed if pulpal damage is suspected to prevent progressive inflammatory resorption [6].

## Case presentation

**Figure 1:**
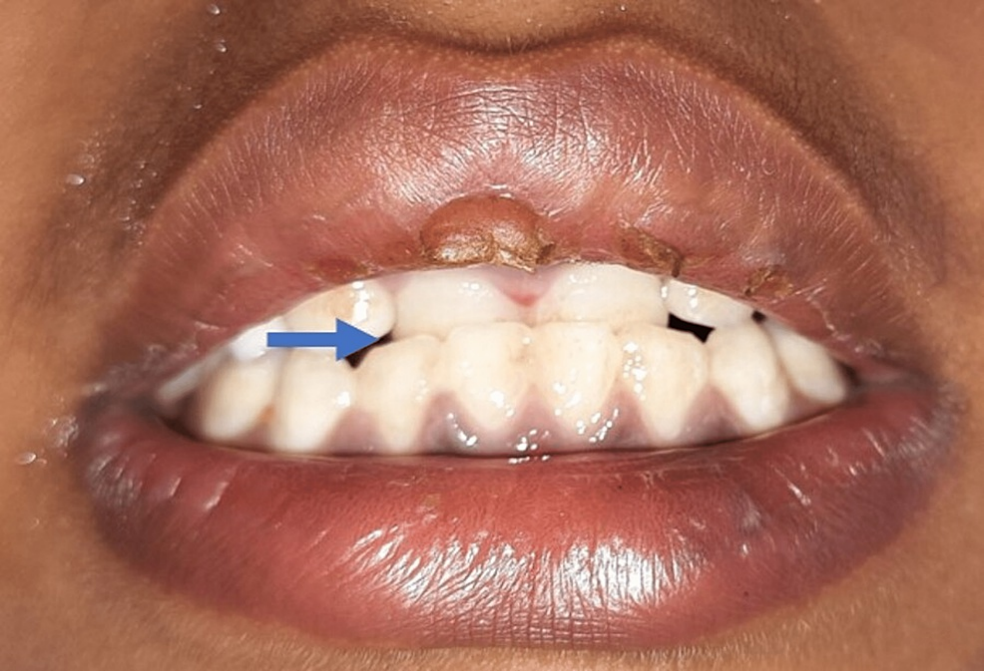
Preoperative image Upper right and left central incisors in crossbite with the lower incisors are depicted (Blue arrow)

**Figure 2:**
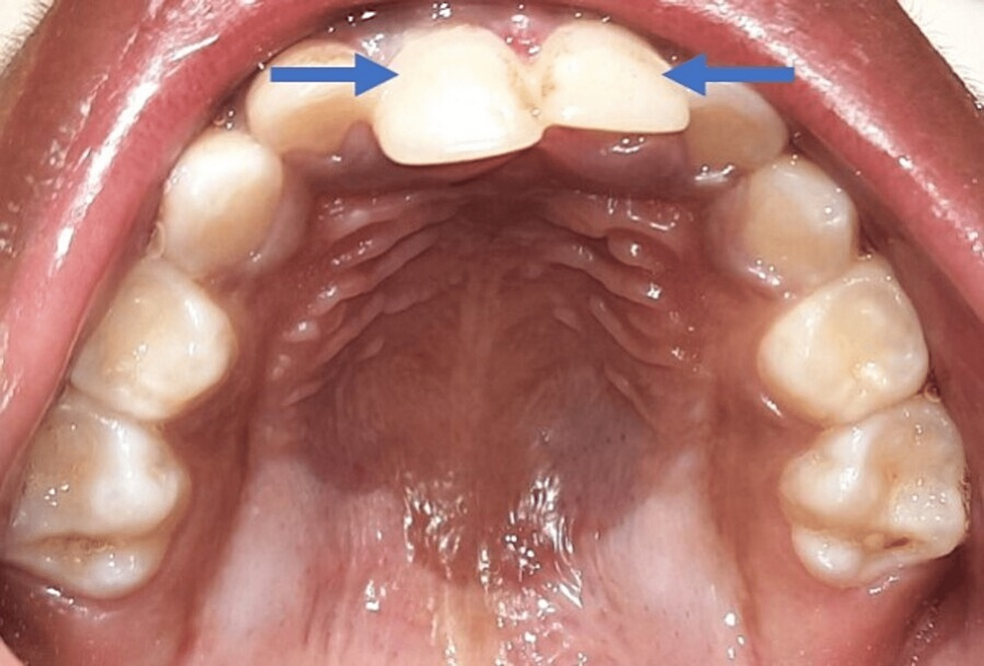
Lateral luxation of the upper right and left central incisors (Blue arrow)

**Figure 3:**
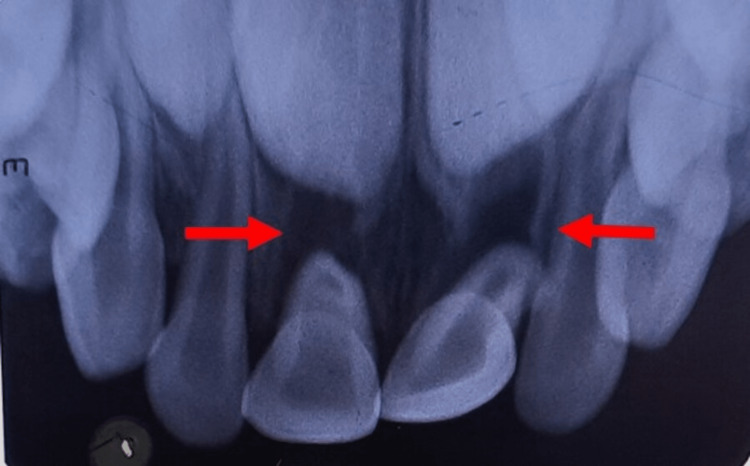
Preoperative intraoral radiograph Palatal displacement of the upper right and left central incisor is seen (Red arrow)

A 5-year-old male patient was referred to the Department of Pediatric and Preventive Dentistry with chief complaints of pain and mobility in the upper front tooth region for 4 days. The patient was apparently alright when he fell from a chair and started experiencing pain in the upper front tooth region of the jaw. There was a history of bleeding from the upper front tooth region; however, no history of unconsciousness or vomiting. On examination, it was seen that the fall had resulted in lateral luxation and palatal displacement of the upper right and left central incisor with grade 2 mobility and crossbite with the lower anterior teeth (Figures 1-2). In the first appointment, the intra-oral periapical radiograph was taken, which showed complete root formation of both teeth, the roots appear shorter due to the palatal displacement of the upper right and left central incisor, and no alveolar fracture was noticed (Figure 3).

**Figure 4:**
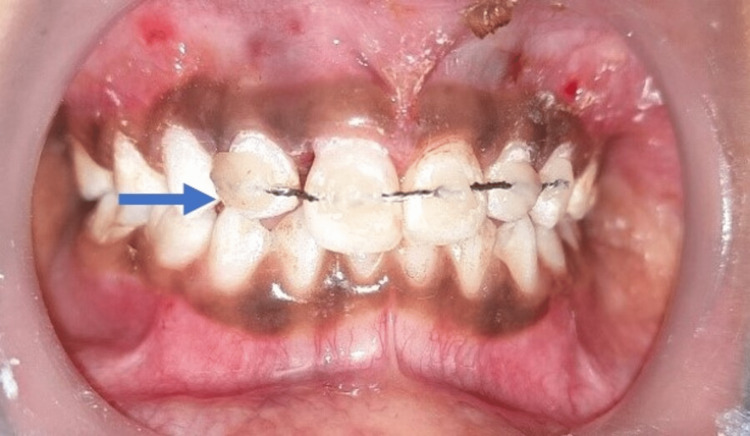
Composite flexible splinting (Blue arrow) Composite flexible splinting was done from the upper right lateral incisors to the left canine

2% lignocaine hydrochloride jelly was applied over the buccal mucosa then 2 ml of local infiltration was administered bilaterally with 2% lignocaine with adrenaline. The upper right and left central incisors were repositioned to their original position by active repositioning using thumb pressure and composite flexible splinting was done to the adjacent unaffected teeth for 4 weeks (Figure 4). Ligature wire was used to make the flexible splint which was twisted twice so the wire would have the desired diameter of 0.4 mm which is ideal for a flexible splint.

**Figure 5:**
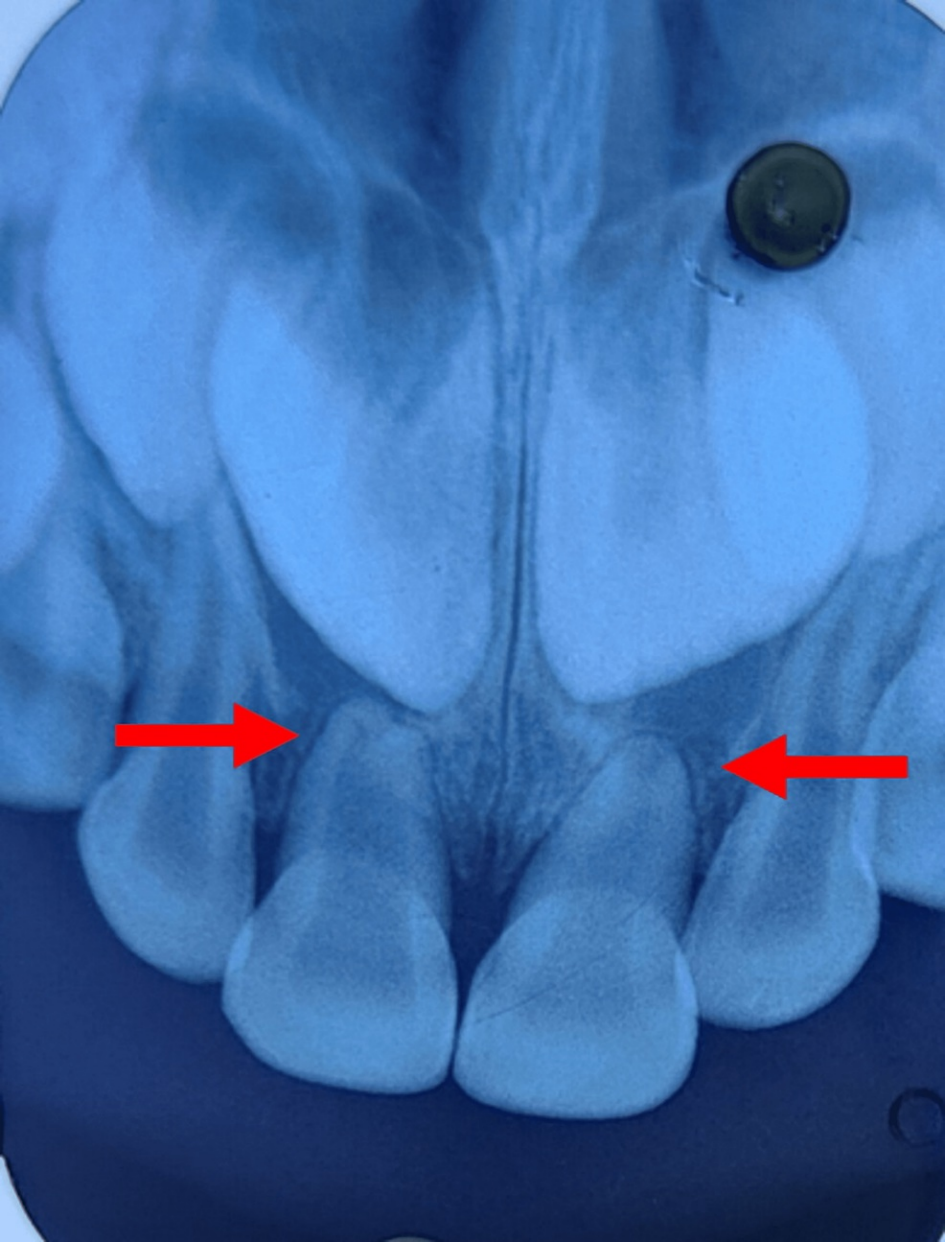
6-month postoperative intraoral periapical radiograph Red arrow depicts the normal root length and periodontal space of the upper right and left central incisors

The antibiotic was prescribed (amoxicillin 125 mg syrup every 8 h for a duration of 5 days). Parents were also instructed on how to maintain oral hygiene to facilitate good healing of the injured tissue. They were asked to use a soft brush for brushing the child’s teeth after every meal which is very important to prevent accumulation of plaque and debris on the teeth. It was also recommended to follow a soft diet for 10-14 days. Seven days after the immediate treatment, the teeth were accessed no abnormality or discoloration was detected. The patient was recalled for follow-up every week for 4 weeks. After 4 weeks, the flexible splint was removed. No mobility was detected after 4 weeks. The patient was also recalled after 8 weeks and 6 months for review. When the patient reported for follow-up after 6 months, a radiographic evaluation was done which showed normal root length and periodontal space (Figure 5).

The parents were also informed to watch out for any complications and return to the clinic if any unfavorable outcome is identified.

## Discussion

Pain management, potential harm to the growing permanent tooth bud, and reducing the likelihood of sequelae are the key goals and factors that are important for the diagnosis and treatment of TDIs in children with primary dentition [7-8]. The TDI therapy for deciduous teeth differs from that for permanent teeth because of the close proximity of the deciduous tooth’s root apex to its permanent successor. Primary teeth can suffer more harm from improper care than from the trauma itself. Treatments that stop the progression of TDI’s sequelae are therefore crucial. The degree of tooth displacement, dental mobility, root formation, and the child’s capacity to handle an emergency should all be considered by the dentist, in accordance with the International Association for Dental Traumatology (IATD) procedures [8-10].

The most common complication of TDI is periapical lesions, pulpectomy can be an essential procedure that will be helpful to revive the health of the teeth that would have otherwise been extracted if not treated. This procedure also stops the spread of illness and puts the tooth back in its proper place in the dental arch [10].

The tooth in question was gently relocated and splinted to the neighboring teeth for 4 weeks in accordance with the most recent IATD protocol for lateral luxated primary teeth. IATD strongly advises splinting despite the fact that there are not much research looking into the subject and the prognosis associated with using splints has not been well examined. Retrospective research on the results of traumatized primary teeth treated with splints was done by Cho et al. [11]. They noted that splinting improved prognosis in root fractures, demonstrating how timely intervention and this kind of conservative treatment can favor a positive result.

It is possible for the neurovascular supply to the pulp to be disrupted when teeth are knocked out by luxation injuries. Periodontal fibers are crushed, and supply channels to the pulp are constrained or compressed. This causes ischemia, which may result in necrosis [12]. Splinting worked well in the reported case, with positive results. As previously mentioned, the deciduous tooth showed no indicators of pulp modification or sensitivity to percussion at the 6-month follow-up, and the crown exhibited no indications of color change.

## Conclusions

Every TDI requests a specific treatment in an assertive and timely manner. When treating a TDI the clinician has to take into consideration the child’s ability to cope with the trauma, the time of exfoliation of the injured primary tooth, and the occlusion which are the most important factors for treatment. In the current case report as both teeth have been saved and are perfectly retained and functional, this reported case may be deemed successful.

## References

[1] Rouhani A, Movahhed T, Ghoddusi J, Mohiti Y, Banihashemi E, Akbari M (2015). Anterior traumatic dental injuries in East Iranian school children: prevalence and risk factors. Iran Endod J.

[2] Glendor U (2008). Epidemiology of traumatic dental injuries-a 12 year review of the literature. Dent Traumatol.

[3] Andreasen JO, Bakland LK, Andreasen FM (2006). Traumatic intrusion of permanent teeth. Part 2. A clinical study of the effect of preinjury and injury factors, such as sex, age, stage of root development, tooth location, and extent of injury including number of intruded teeth on 140 intruded permanent teeth. Dent Traumatol.

[4] Kharpate S, Rathi N, Gomase PV, Baliga S, Thosar N (2020). Appraisal of awareness and attitude of school teachers towards emergency management of dental trauma and tooth avulsion replantation. J Evolution Med Dent Sci.

[5] McTigue DJ (2013). Overview of trauma management for primary and young permanent teeth. Dent Clin North Am.

[6] Diangelis AJ, Andreasen JO, Ebeleseder KA (2012). International Association of Dental Traumatology guidelines for the management of traumatic dental injuries: 1. Fractures and luxations of permanent teeth. Dent Traumatol.

[7] Flores MT (2002). Traumatic injuries in the primary dentition. Dent Traumatol.

[8] Faria LV, Chaves HG, Borges Silva EA, Antunes LS, Antunes LA (2020). Minimally invasive treatment of an extruded deciduous tooth - Case report. Dent Traumatol.

[9] Antunes LA, Lemos HM, Milani AJ, Guimarães LS, Küchler EC, Antunes LS (2020). Does traumatic dental injury impact oral health-related to quality of life of children and adolescents? Systematic review and meta-analysis. Int J Dent Hyg.

[10] Abreu MG, Milani AJ, Fernandes TD, Gomes CC, Antunes LS, Antunes LA (2020). Dental trauma in primary dentition, its effect on permanent successors and on Oral Health-Related Quality of Life: a 4-year follow-up case report. Int J Burns Trauma.

[11] Cho WC, Nam OH, Kim MS, Lee HS, Choi SC (2018). A retrospective study of traumatic dental injuries in primary dentition: treatment outcomes of splinting. Acta Odontol Scand.

[12] Lam R (2016). Epidemiology and outcomes of traumatic dental injuries: a review of the literature. Aust Dent J.

